# Early-life stress impairs recognition memory and perturbs the functional maturation of prefrontal-hippocampal-perirhinal networks

**DOI:** 10.1038/srep42042

**Published:** 2017-02-07

**Authors:** Samuel A. J. Reincke, Ileana L. Hanganu-Opatz

**Affiliations:** 1 Developmental Neurophysiology, Institute of Neuroanatomy, University Medical Center Hamburg-Eppendorf, 20251 Hamburg, Germany

## Abstract

Early life exposure to stressful situations impairs cognitive performance of adults and contributes to the etiology of several psychiatric disorders. Most of affected cognitive abilities rely on coupling by synchrony within complex neuronal networks, including prefrontal cortex (PFC), hippocampus (HP), and perirhinal cortex (PRH). Yet it remains poorly understood how early life stress (ELS) induces dysfunction within these networks during the course of development. Here we used intermittent maternal separation during the first 2 postnatal weeks to mimic ELS and monitored the recognition memory and functional coupling within prefrontal-hippocampal-perirhinal circuits in juvenile rats. While maternally-separated female rats showed largely normal behavior, male rats experiencing this form of ELS had poorer location and recency recognition memory. Simultaneous multi-site extracellular recordings of network oscillations and neuronal spiking from PFC, HP, and PRH *in vivo* revealed corresponding decrease of oscillatory activity in theta and beta frequency bands in the PFC of male but not female rats experiencing maternal separation. This deficit was accompanied by weaker cross-frequency coupling within juvenile prefrontal-hippocampal networks. These results indicate that already at juvenile age ELS mimicked by maternal separation induces sex-specific deficits in recognition memory that might have as underlying mechanism a disturbed communication between PFC and HP.

Childhood adversity has been associated with one third of psychiatric disorders at adulthood^1^. Understanding the causal relationship between a history of early maltreatment and subsequent disease requires a mechanistic elucidation of stress action that is difficult to achieve in humans^2^. In animal models, adverse or deprived caregiving has been identified as a highly potent and ethologically relevant early life stressor that contributes to emotional and cognitive dysfunction throughout life^3^,^4^. Maternal separation impairs aversive conditioning, preparing the animals to respond more cautiously toward fear signals in their environment^5^,^6^. Moreover, it decreases the adult performance in inhibitory learning and spatial/relational memory^7^,^8^. Being free of test-related stress components and using the animal’ inherited preference for novelty^9^, recognition memory has been investigated to monitor the long-term consequences of maternal deprivation in rodents^10^,^11^,^12^. It has been shown that adult rats experiencing separation from the mother during the first two postnatal weeks showed poorer ability to recognize new objects.

Recognition memory is one of the first cognitive abilities maturating during juvenile development. It relies on a well characterized neuronal network including PFC, HP and PRH^13^,^14^,^15^. While recognition of a novel object critically depends on PRH, the object-in-place and recency recognition have been proposed to result from interactions between PFC and HP^13^,^14^,^15^. Recently, the complex dynamics of these interactions have been exemplified for the directed prefrontal-hippocampal coupling in context-guided memory^16^. The long-range coupling within prefrontal-hippocampal networks is initiated during the first postnatal week with a prominent oscillatory drive in theta frequency range from HP to PFC and feedback communication via thalamic nuclei^17^,^18^. With ongoing maturation the directed interactions are replaced by mutual coupling by synchrony of the two brain areas^17^. Even if transient, the unidirectional prefrontal-hippocampal communication during early development seems to be relevant for the maturation of recognition memory^19^. The detrimental influence of maternal separation on the ability to recognize new objects might indicate that ELS interferes with the correct wiring of prefrontal-hippocampal networks. Disruption of cortical excitability, dendritic atrophy and spine loss have been proposed as synaptic and cellular substrate of ELS-induced cognitive dysfunction at adulthood^10^,^20^,^21^,^22^,^23^,^24^. However, it is still fully unknown whether and at which developmental time point the wiring and function of neuronal networks is affected by ELS. Maternal separation has been reported to affect not only the adult but also the juvenile behavior and some deficits, such as working-memory impairment after ELS, are present solely during adolescence^25^.

Here we combine electrophysiology *in vivo* with behavioral testing to assess the consequences of transient maternal separation during the first two postnatal weeks on recognition memory as well as on activity patterns and coupling by synchrony within prefrontal-hippocampal-perirhinal networks of juvenile rats. We show that decreased oscillatory activity in the PFC and abnormal prefrontal-hippocampal coupling are present already at juvenile age in male rats. This dysfunction correlates with poorer object location and recency recognition. These findings provide mechanistic insights into the developmental dynamics of ELS-induced circuit dysfunction.

## Results

### Early-life stress impairs recognition memory and alters exploratory behavior of juvenile rats

While emotional and cognitive dysfunction of adult rats with a history of ELS have been extensively documented, the ontogeny of these behavioral deficits remains, however, poorly understood. We used different recognition memory paradigms to decide whether already at early stages of juvenile development, the cognitive behavior of rats maternally deprived during the first postnatal weeks is affected.

In a first step, all rats were investigated in their somatic development from P3 to P16 to control for any possible effects of ELS on overall growth and metabolism. All rats showed progressive increase in body weight from 11.7 ± 0.31 g (male), 11.3 ± 0.30 g (female) in control group (CON) (n = 22) and 11.7 ± 0.28 g (male), 11.3 ± 0.26 g (female) in ELS group (n = 22) at P3 [two-way ANOVA, group: F(1,40) = 0.012, p = 0.91; sex: F(1,40) = 1.90, p = 0.18; group × sex: F(1,40) = 0.002, p = 0.96] to 52.2 ± 1.56 g (male), 49.8 ± 1.18 g (female) and 49.6 ± 1.42 g (male), 47.4 ± 1.73 g (female) respectively, at P16 [two-way ANOVA, group: F(1,40) = 2.88, p = 0.10; sex: F(1,40) = 2.31, p = 0.14; group × sex: F(1,40) = 0.002, p = 0.97]. Neither sex-specific nor group-specific differences in weight gain were detected.

**Figure 1:**
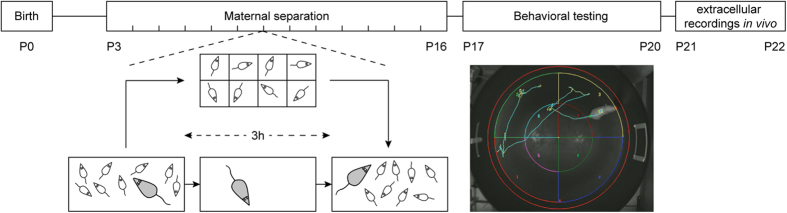
Schematic representation of the experimental protocol. Maternal separation as model of ELS was performed for 2 weeks from P3 to 16. Pups were daily separated from mother and siblings for 3 hours/day and placed individually in temperature- (34 °C) and humidity-controlled cages. Behavioral testing of locomotion (open field in a circular arena), anxiety and recognition memory at P17–20 was followed by recordings of LFP and MUA *in vivo* from PFC, HP and PRH of P21–22 rats. Day of birth was P0.

**Figure 2:**
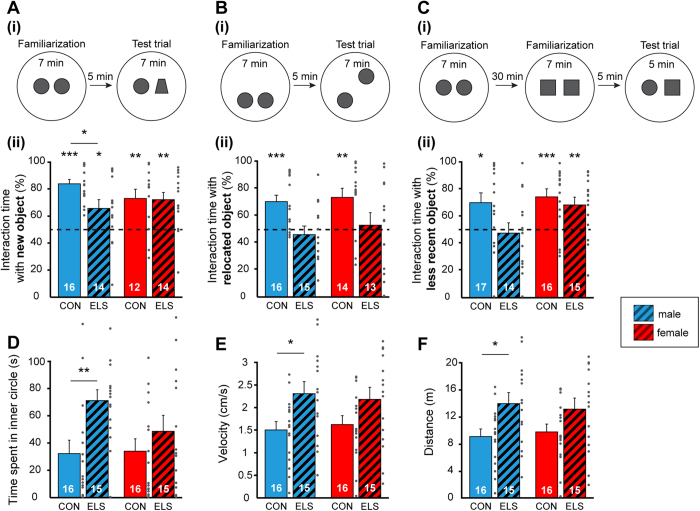
Exploratory behavior and recognition memory of controls and maternally-separated rats at juvenile age. (**A**) Novel object recognition. (i) Schematic diagram of the NOR protocol. (ii) Bar diagram displaying the relative interaction time spent by controls (CON) and maternally-separated rats (ELS) with the novel object. (**B**) Same as (**A**) for object location recognition task. (**C**) Same as (**A**) for recency recognition task. For (**A**), (**B**) and (**C**) dotted lines correspond to chance level. (**D**) Bar diagram displaying the mean time spent in the inner circle during open field task by CON and ELS rats. (**E,F**) Bar diagram displaying the mean velocity and running distance of CON and ELS rats during open field task. Data for male and female are shown in blue and red, respectively. Data are displayed as mean ± SEM. Grey dots correspond to individual values and the number of investigated animals is marked on each bar. (*)p < 0.05, (**)p < 0.01 and (***)p < 0.001.

In a second step, we tested juvenile CON and ELS rats for their ability to recognize (i) new objects in a novel object recognition test (NOR) at P18, (ii) the new position of an object in an object location recognition test (OLR) at P19, and (iii) the temporal dynamics of getting familiar with two objects in a recency recognition test (RR) at P20. For this, a custom-designed arena and objects of different size, texture, and color were used (Fig. 1). In line with our previous data^19^, we initiated the investigation after full maturation of sensory and motor abilities required for processing of novelty (P14). During the familiarization trial for NOR all rats, independently of their group, spent equal time investigating the two objects placed in the arena. During test trial male CON [t-test, 84.00 ± 3.23%, t(15) = 10.53, p < 0.001] and ELS animals [t-test, 66.06 ± 6.56%, t(13) = 2.45, p = 0.029] as well as female CON [t-test, 73.27 ± 6.90%, t(11) = 3.37, p = 0.006] and ELS animals [t-test, 72.08 ± 5.78%, t(13) = 3.82, p = 0.002] spent significantly longer time with the novel object (Fig. 2A). However male ELS animals showed a poorer performance and spent significantly less time with the new object, compared to male CON animals [t-test, t(28) = −2.55, p = 0.016, two-way ANOVA, group: F(1,52) = 2.91, p = 0.09; effect of sex: F(1,52) = 0.18, p = 0.68; group × sex: F(1,52) = 2.23, p = 0.14]. Thus, ELS does not impair the novel object recognition at juvenile age, though male ELS animals showed weaker performance.

During OLR task rats experienced a position change of one of the familiar objects in the testing arena. Both male and female ELS pups did not differentiate between the familiar and novel position of the objects [t-test, male: 46.24 ± 6.07%, t(14) = −0.62, p = 0.55; female: 53.11 ± 9.28%, t(12) = 0.34, p = 0.74], whereas CON animals, male and female spent longer time exploring the object at new position [t-test, male: 70.60 ± 4.89%, t(15) = 4.21, p < 0.001; female: 73.76 ± 6.71%, t(13) = 3.54, p = 0.004] (Fig. 2B). Comparing the discrimination ratio a significant group, but not sex effect could be detected [two-way ANOVA, group: F(1,54) = 11.24, p = 0.001; sex: F(1,54) = 0.56, p = 0.46; group × sex: F(1,54) = 0.08, p = 0.78].

During RR task rats had to process temporal information by recognizing the set of objects which they most recently got familiar with. Male CON rats spent significantly longer time with the object from the first familiarization trial [t-test, 70.09 ± 7.14%, t(16) = 2.81, p = 0.013], whereas male ELS rats explored both objects equally long [t-test, 47.68 ± 7.63%, t(13) = −0.30, p = 0.77] (Fig. 2C). In contrast, female CON and ELS rats recognized the object they most recently interacted with and spent significantly longer time with the object from the first familiarization trial [t-test, CON: 74.55 ± 5.88%, t(15) = 4.17, p < 0.001; ELS: 68.53 ± 5.80%, t(14) = 3.20, p = 0.006]. Comparing the discrimination ratio a significant group, but not sex effect could be detected [two-way ANOVA, group: F(1,58) = 4.54, p = 0.037; sex: F(1,58) = 3.59, p = 0.063; group × sex: F(1,58) = 1.51, p = 0.22].

The incapacity of rats experiencing ELS to achieve normal OLR and RR may result from their poorer motor abilities and/or enhanced anxiety when interacting with the objects. However, the opposite was the case. During the open field task performed at P17, male ELS rats spent significantly longer time in the inner circle when compared to CON animals [t-test, CON: 32.26 ± 9.85 s, ELS: 71.09 ± 7.94 s, t(29) = −3.05, p = 0.005], suggesting that they have reduced anxiety (Fig. 2D). Moreover, these maternally-separated rats were hyperactive, their velocity (2.31 ± 0.27 cm/s) and distance (13.85 ± 1.60 m) covered in the open field being significantly [t-test, t(29) = −2.49, p = 0.019, t(29) = −2.49, p = 0.019] augmented when compared with male CON rats (9.03 ± 1.12 m, 1.51 ± 0.19 cm/s) (Fig. 2E,F). In contrast, female CON and ELS animals spent equal time in the inner circle [t-test, CON: 34.08 ± 8.95 s, ELS: 48.60 ± 11.65 s, t(29) = −1.0, p = 0.33] (Fig. 2D) and no differences in the level of motor activity in the arena were detected [t-test, distance, CON: 9.73 ± 1.17 m, ELS: 13.04 ± 1.61 m, t(29) = −1.68, p = 0.10; velocity, CON: 1.62 ± 0.19 cm/s, ELS: 2.17 ± 0.27 cm/s, t(29) = −1.68, p = 0.10] (Fig. 2E,F). Significant group-specific effects were detected for distance [two-way ANOVA, F(1,58) = 8.68, p = 0.005], velocity [two-way ANOVA, F(1,58) = 8.68, p = 0.005] and the time spent in the inner circle [two-way ANOVA, F(1,58) = 7.59, p = 0.008], whereas sex- and group × sex-specific effects remained below significance level.

Taken together, these results indicate that ELS impairs the cognitive and emotional abilities of male and to a lesser extent of female rats already at juvenile age. While male rats have poorer spatial and temporal recognition memory as well as hyperactivity and decreased anxiety, female rats have milder behavioral outcome of early adversity, showing solely disrupted spatial processing.

### Early-life stress perturbs the coordinated patterns of activity in the juvenile prefrontal cortex, but not hippocampus and perirhinal cortex

The poorer performance of juvenile ELS rats in recognition memory tasks may reflect the abnormal function of underlying neuronal networks centered on the PFC, HP and PRH. To test this hypothesis, we monitored the patterns of network activity in all three areas by simultaneous extracellular recordings of local field potential (LFP) and multiple unit activity (MUA) from the prelimbic subdivision of the PFC (PL), which is known to tightly interact with the HP during development^17^, the hippocampal CA1 area and the PRH of urethane-anesthetized P21–22 CON (male, n = 10; female, n = 11) and ELS animals (n = 9, n = 8, respectively) *in vivo*. The rats previously underwent behavioral testing.

**Figure 3:**
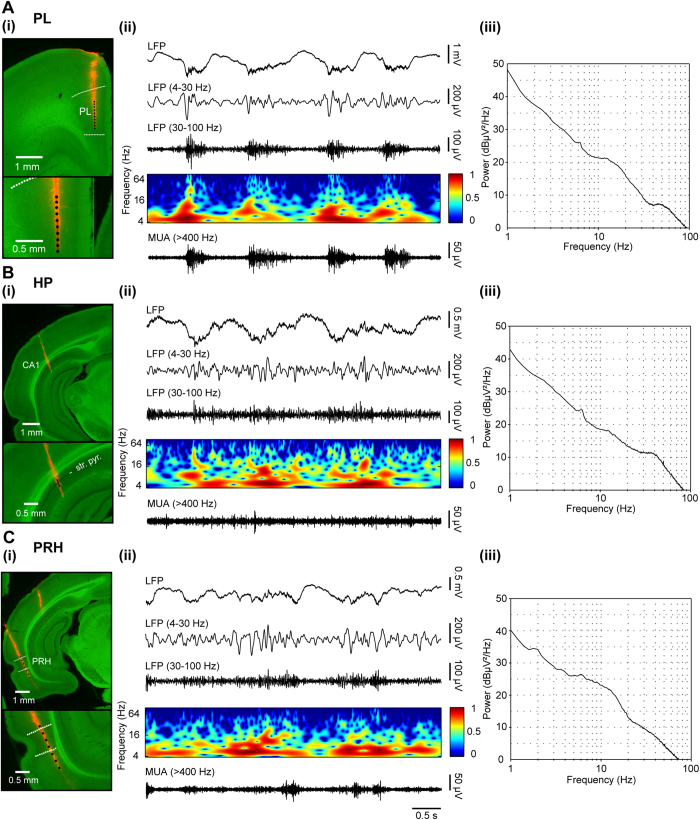
Patterns of oscillatory activity in the PL, HP and PRH of juvenile rats. (**A**) Patterns of oscillatory activity in the PL. (i) Top, digital photomontage reconstructing the location of the DiI-labeled recording electrode (orange) in the PL of a Nissl-stained 100 μm-thick coronal section (green). Bottom, the location of the electrode displayed at higher magnification. Dots mark the 16 recording sites covering the PL. (ii) Extracellular LFP recording of the intermittent oscillatory activity in the PL of a P21 rat (top) accompanied by the color-coded wavelet spectrum and the corresponding MUA after 200 Hz highpass filtering (bottom). (iii) Logarithmic power spectrum of the network activity from one whole recording of a P21 rat for PL illustrating peaks in theta (6.5 Hz), beta (15 Hz) and gamma band (45 Hz). (**B**) Same as for (**A**) for HP from the same rat. (**C**) Same as (**A**) for PRH from the same rat.

At juvenile age the network activity of all cortical areas switches from discontinuous theta-beta discharges to adult-characteristic continuous patterns, but still differs from the latter in many aspects^17^. Both CON and ELS rats showed similar patterns of oscillatory activity, which correspond to the previously reported sleep-like rhythms mimicked by urethane anesthesia^26^,^27^,^28^. They were most prominent in the PL, thus the prelimbic recordings were selected as reference for their classification. As previously reported^17^,^28^, the dominant network pattern present in all rats was the large-amplitude irregular activity, which was characterized by prominent slow potential fluctuations with no rhythmicity. Low-amplitude oscillatory activity within theta, beta, and gamma frequency range superimposed the ground irregular discharge, as reflected by small, but consistent power peaks within these three bands (Fig. 3). Taking into account that these frequencies have been identified as having different functions for the juvenile network communication^17^, the further analysis was separately conducted for three bands: theta (4–12 Hz), beta (12–30 Hz) and gamma (30–100 Hz).

**Figure 4:**
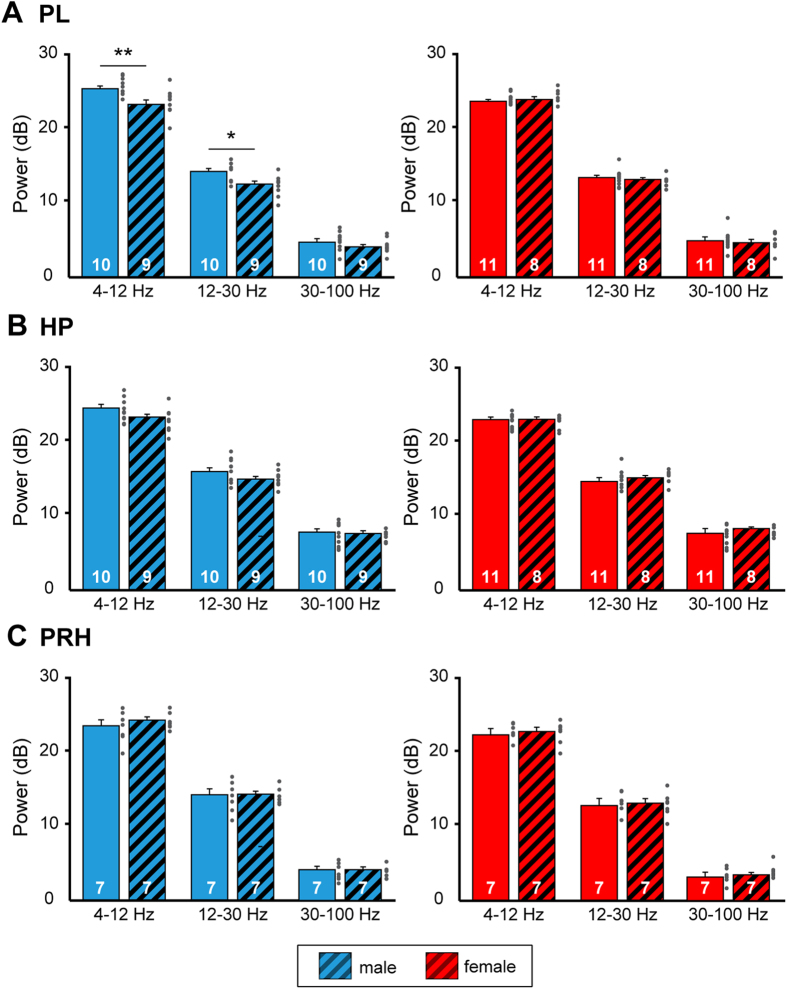
Consequences of maternal deprivation on juvenile network activity. (**A**) Bar diagram displaying the mean power of oscillatory activity in theta, beta and gamma band recorded from the PL of male (blue) and female (red) CON and ELS rats. (**B**) Same as (**A**) for oscillatory activity in HP. (**C**) Same as (**B**) for oscillatory activity in PRH. Data are displayed as mean ± SEM. Grey dots correspond to individual values and the number of investigated animals is marked on each bar. (*)p < 0.05 and (**)p < 0.01.

Male rats experiencing ELS showed significantly lower power of theta (p = 0.009, corrected significance level = 0.016) and beta (p = 0.01) oscillatory activity in PL when compared with CON animals (Fig. 4A, Table 1). The gamma power of their network activity was not affected by maternal separation (p = 0.16). In contrast, the oscillatory activity of female rats at juvenile age was not affected by ELS, the power in theta, beta, and gamma bands being similar with those averaged for CON rats (Table 1). These results demonstrate a group × sex effect for theta band activity [two-way ANOVA, theta: group × sex: F(1,34) = 7.38, p = 0.01]. The juvenile oscillatory activity in HP and PRH did not differ between CON and ELS rats (Fig. 4B,C, Table 1). The power of theta, beta and gamma oscillations was comparable in the two groups and no sex-specific differences were detected.

These results highlight a sex- and brain area-specific dysfunction of juvenile neuronal networks after ELS and identify the PFC of juvenile males as a highly vulnerable area.

### Early-life stress perturbs the communication within prefrontal-hippocampal-perirhinal networks at juvenile age

Taking into account the tight coupling between PFC, HP and PRH during recognition memory tasks, the lower level of network activation in the PFC of male ELS rats might impact the behaviorally-relevant functional communication between the three brain areas. To test this hypothesis, we assessed the synchrony and directionality of interactions between PL and HP, PL and PRH as well as HP and PRH in juvenile CON and ELS rats.

**Figure 5:**
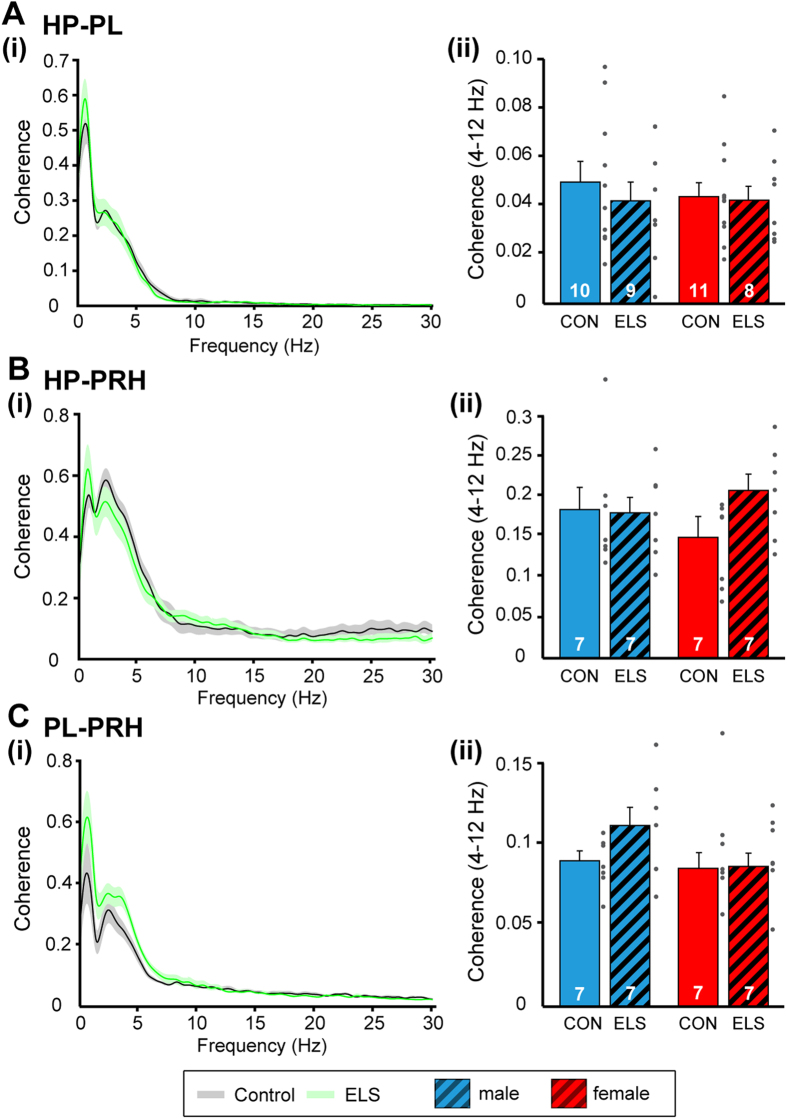
Consequences of maternal deprivation on network synchrony. (**A**) Coupling by synchrony within prelimbic-hippocampal networks. (i) Averaged coherence spectrum for male CON (grey) and ELS (green) rats showing similar peaks at ~1 Hz and between 3–6 Hz. Female CON and ELS showed similar peaks. (ii) Bar diagram displaying the coherence in the theta band (4–12 Hz) averaged for all male (blue) and female (red) CON and ELS rats. (**B**) Same as for (**A**) for coherence between HP and PRH. (**C**) Same as for (**A**) for coherence between PL and PRH. For all spectra, the transparent area corresponds to SEM. For bar diagrams, data are displayed as mean ± SEM. Grey dots correspond to individual values and the number of investigated animals is marked on each bar.

In a first step, we monitored the synchrony between pairs of regions by calculating the coherence. Mirroring the prominent slow large-amplitude irregular activity present in all investigated areas, the first coherence maximum was detected for frequencies around 1 Hz. A second peak of coherence couples all pairs of regions at frequencies within 3–6 Hz (Fig. 5), the range that has been identified as substrate of long-range communication within the juvenile brain^17^,^28^. In line with their dense anatomical connectivity^29^, PRH and HP had the strongest functional coupling by synchrony (male: 0.18 ± 0.03, female: 0.14 ± 0.02), whereas the coherence between PL and HP as well as between PL and PRH were smaller (male: 0.05 ± 0.01, female: 0.04 ± 0.01; male: 0.09 ± 0.01, female: 0.08 ± 0.01, respectively). Thus, for none of investigated pairs of regions the coherence in theta frequency range significantly differed between juvenile CON and ELS rats. Moreover, no group- or sex-dependent differences in the coupling within prelimbic-hippocampal-perirhinal networks were detected [two-way ANOVA, HP-PRH: group: F(1,24) = 1.70, p = 0.20; sex: F(1,24) = 0.11, p = 0.74; group × sex: F(1,24) = 2.19, p = 0.15; HP-PL: group: F(1,34) = 0.38, p = 0.54; sex: F(1,34) = 0.16, p = 0.69; group × sex: F(1,34) = 0.17, p = 0.68; PL-PRH: group: F(1,24) = 0.74, p = 0.39; sex: F(1,24) = 0.14, p = 0.71; group × sex: F(1,24) = 1.23, p = 0.28] (Fig. 5).

**Figure 6:**
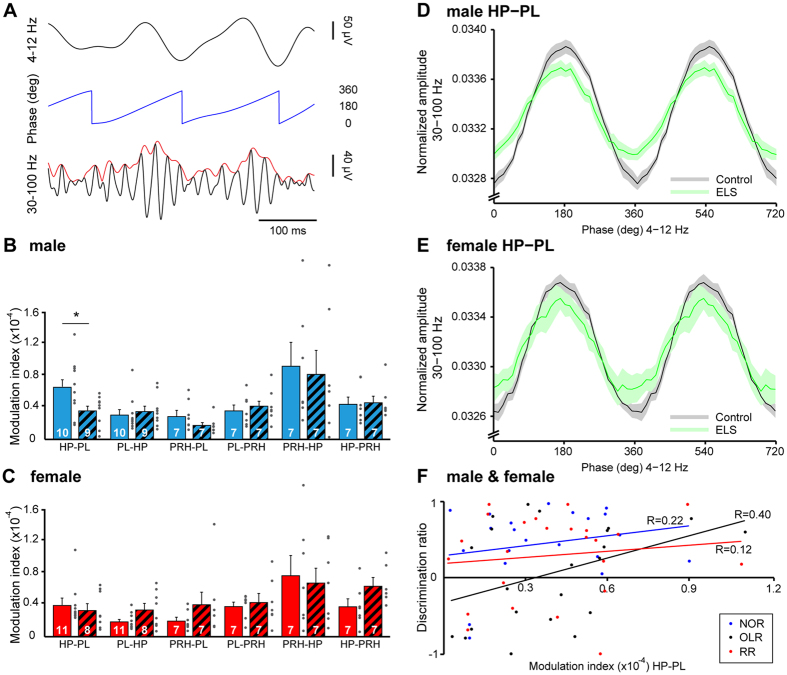
Consequences of maternal separation on theta-gamma cross-frequency-coupling (CFC) within prelimbic-hippocampal-perirhinal networks. (**A**) Schematic diagram of the parameters necessary for CFC calculation: phase (blue, middle) of theta oscillations (top) and amplitude (red, bottom) of simultaneously recorded gamma oscillations. (**B**) Bar diagram displaying the CFC modulation indices calculated for pairs of regions in male CON (blue solid) and ELS rats (blues dashed). The theta phase corresponds to the firstly named region and the gamma-amplitude to the secondly named one. (**C**) Same as (**B**) for female CON and ELS rats. (**D**) Line plot displaying the phase-amplitude coupling of hippocampal theta phase and prelimbic gamma amplitude averaged for male CON (black) and ELS (green). The transparent area corresponds to SEM. (**E**) Same as (**D**) for female CON and ELS. (**F**) Scatter plot displaying the relationship between the hippocampal-prelimbic CFC and the behavioral performance in NOR, OLR and RR. The regression lines are displayed in the corresponding color (blue for NOR, black for OLR and red for RR) and the regression coefficients (R) are indicated. For bar diagrams, data are displayed as mean ± SEM. Grey dots correspond to individual values and the number of investigated animals is marked on each bar. (*)p < 0.05.

Besides coupling by synchrony, neuronal networks efficiently and precisely communicate by establishing interactions between frequency bands, a phenomenon termed as cross-frequency coupling (CFC)^30^. In particular, phase-amplitude CFC, which describes the statistical dependence between the phase of a low frequency oscillation (e.g. theta) and the amplitude of the fast rhythm (e.g. gamma), integrates activity across different spatial and temporal scales. To decide whether theta oscillations coordinate gamma-band spike-based computations of local networks, we calculated theta-gamma CFC within PL, HP and PRH, as well as between pairs of regions (Fig. 6A). The strength of CFC was region specific [three-way ANOVA, effect of region: F(261,8) = 22.16, p < 0.001]. The highest intra-region phase-amplitude CFC was found in PL and PRH with modulation index (MI) values of 0.16 × 10^−3^ ± 0.36 × 10^−4^ and 0.13 × 10^−3^ ± 0.25 × 10^−4^ (male controls), respectively. Inter-area CFC was rather weak but significant when compared with shuffled data [t-test, HP-PL, t(18) = 5.52, p < 0.001; PRH-HP, t(12) = 2.99, p = 0.01] (Fig. 6B,C). Prominent modulation of gamma amplitude in PL by the theta drive from HP was present in male (0.66 × 10^−4^ ± 0.11 × 10^−4^), and in female (0.38 × 10^−4^ ± 0.76 × 10^−5^) CON rats at juvenile age. The prelimbic gamma discharge was timed at the peak of theta cycle in HP (Fig. 6D,E). Moreover, gamma activity in the HP was coordinated by perirhinal theta in both males and females as shown by the high MI (males: 0.93 × 10^−4^ ± 0.31 × 10^−4^, females: 0.77 × 10^−4^ ± 0.26 × 10^−4^).

ELS selectively affected the inter-area CFC in juvenile rats. While the hippocampal-perirhinal coupling was indistinguishable in male ELS rats (0.83 × 10^−4^ ± 0.31 × 10^−4^), the modulation of gamma activity in PL by theta oscillations in HP significantly [t-test, t(17) = 2.28, p = 0.036] decreased (0.36 × 10^−4^ ± 0.65 × 10^−5^) without changing the phase of coupling (Fig. 6B,D), whereas female rats did not show a significant change after ELS [t-test, t(17) = 0.48, p = 0.64] (Fig. 6C). However, the two-way ANOVA revealed a significant group effect [two-way ANOVA, group: F(34,1) = 4.19, p = 0.0485, sex: F(34,1) = 3.03, p = 0.09, group × sex: F(34,1) = 2.05, p = 0.16]. To test whether these findings correlate to the behavioral outcome we calculated the Pearson’s correlation between the discrimination ratio during the test phase of NOR, OLR, and RR and the MI of CFC (hippocampal theta phase to prelimbic gamma amplitude) for all investigated rats (males and females). The CFC weakly correlated with the performance in OLR task (regression coefficient (R) = 0.40, p = 0.06, n = 22), but not with NOR (R = 0.22, p = 0.35, n = 21) and RR (R = 0.12, p = 0.56, n = 24) performance (Fig. 6F).

**Figure 7:**
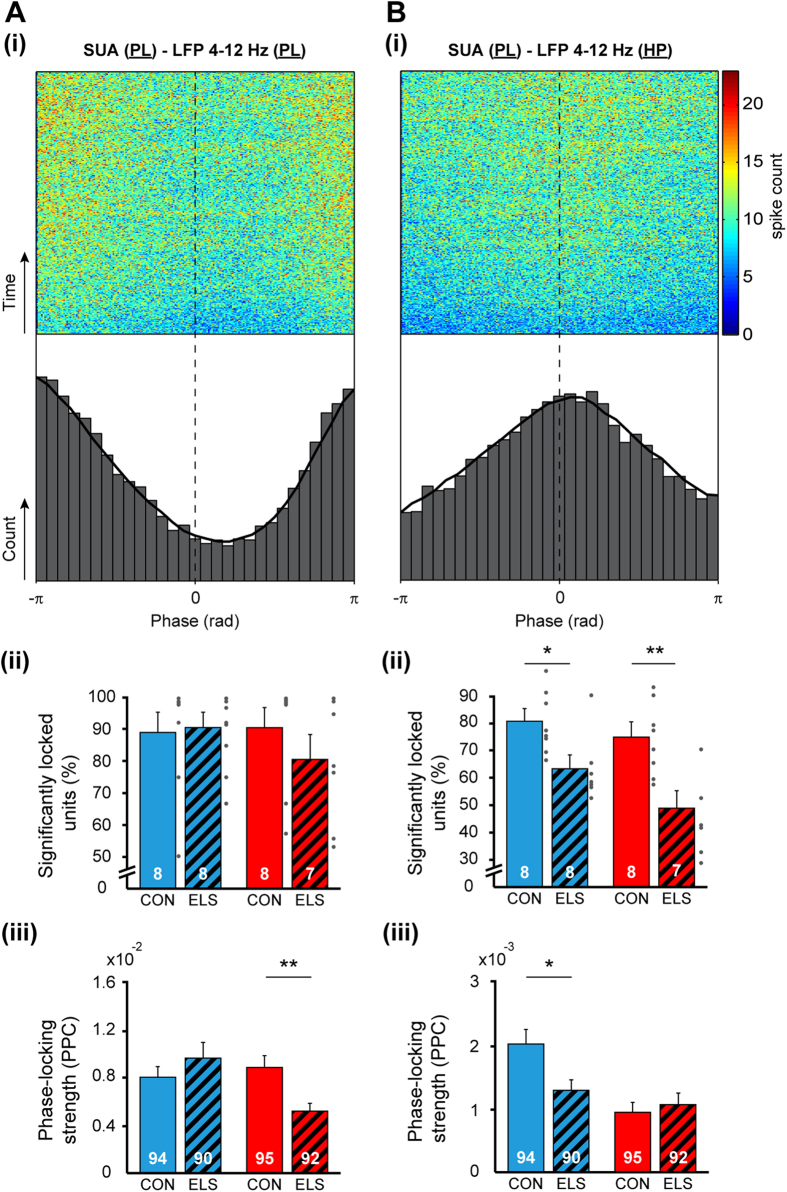
Consequences of maternal separation on spike timing within prelimbic-hippocampal networks. (**A**) Phase locking of prelimbic SUA to the phase of theta oscillations in PL of male CON. Female CON showed same distribution. (i) Top, spike trains of all locked cells from male CON rats were aligned to the trough of a theta cycle and summed up over recording time (y-axis). Bottom, the resulting histogram showing the distribution of spikes over theta cycle. (ii) Bar diagram displaying the relative number of significantly locked cells to theta in PL when averaged for all CON and ELS rats. (iii) Bar diagram showing the strength of phase locking assessed by pairwise phase consistency analysis of all recorded units. (**B**) Same as for (**A**) but for phase locking of SUA in PL to theta in HP. Data are displayed as mean ± SEM. Grey dots correspond to individual values and the number of investigated animals (ii)/cells (iii) is marked on each bar. (*)p < 0.05 and (**)p < 0.01.

In line with the theta-gamma coupling in prelimbic-hippocampal networks, theta in HP has been reported to time the firing of prelimbic neurons both during development and at adulthood^31^. To test whether the phase locking of prelimbic neurons to hippocampal theta is disturbed after maternal separation, we clustered single unit activity (SUA) in PL (CON males: 94 neurons, CON females: 95 neurons; ELS males: 90 neurons, ELS females: 92) and calculated the phase locking of individual firing to theta activity in PL and HP (Fig. 7). In relationship to network oscillations in PL, prelimbic SUA was locked to the trough (180°) of theta oscillation and the fraction of significantly locked cells was similar in CON (males: 88.9 ± 6.3%, females: 90.5 ± 6.3%) and ELS (males: 90.7 ± 4.6%, females: 80.5 ± 7.8%) rats (Fig. 7Ai). Taking into account that ELS led to significant changes of firing rates [males: CON: 2.35 ± 0.23 Hz, ELS: 1.75 ± 0.17 Hz, t-test, t(182) = 2.11, p = 0.036; females: CON: 2.96 ± 0.24 Hz, ELS: 1.60 ± 0.16 Hz, t-test, t(185) = 4.65, p < 0.001; two-way ANOVA: effect of group: F(370,1) = 23.01, p < 0.001], we assessed the locking strength by calculating the pairwise phase consistency (PPC) that is independent of spike number^32^,^33^. The locking strength decreased in females [CON: 0.88 × 10^−2^ ± 0.10 × 10^−2^, ELS: 0.52 × 10^−2^ ± 0.07 × 10^−2^, t-test, t(185) = 3.03, p = 0.003], but not in males [CON: 0.79 × 10^−2^ ± 0.10 × 10^−2^, ELS: 0.96 × 10^−2^ ± 0.13 × 10^−2^, t-test, t(182) = −1.00, p = 0.32] (Fig. 7Aiii). These results were confirmed by a significant group × sex effect [two-way ANOVA: F(367,1) = 6.73, p = 0.01]. In contrast, in relationship to network oscillations in HP, SUA was locked to the peak of the theta oscillation (Fig. 7Bi). ELS significantly reduced the number of cells timed by the theta rhythm from 80.62 ± 4.11% in CON to 63.35 ± 4.26% [t-test, t(14) = 2.91, p = 0.01] in maternally separated males and from 74.9 ± 4.7% in CON to 48.9 ± 6.4% [t-test, t(13) = 3.32, p = 0.005] in maternally separated females [two-way ANOVA, effect of group: F(27,1) = 19.77, p < 0.001] (Fig. 7Bii). Correspondingly, the locking strength decreased in males from 2.0 × 10^−3^ ± 0.20 × 10^−3^ in CON to 1.3 × 10^−3^ ± 0.20 × 10^−3^ in ELS [t-test, t(182) = 2.56, p = 0.01]. However in females the locking strength remained unaffected [CON: 0.90 × 10^−3^ ± 0.10 × 10^−3^, ELS: 1.10 × 10^−3^ ± 0.20 × 10^−3^, t-test, t(185) = −0.55, p = 0.58] (Fig. 7Biii).

These data indicate that prelimbic-hippocampal communication of juvenile rats experiencing ELS is subtly altered when compared with age-matched controls.

## Discussion

In the present study, we combined behavioral testing with electrophysiology *in vivo* from urethane-anesthetized rats to decide how adversity during early life perturbs the functional development of neuronal networks and related behavioral performance. We used transient maternal separation during the first two postnatal weeks as a well characterized model of ELS. We showed that the ontogeny of cognitive performance at juvenile age was severely affected in males and to a lesser extent, in female rats with a history of ELS. These deficits correlate with lower theta-beta power of oscillatory activity in the prelimbic subdivision of PFC, abnormal theta-gamma entrainment within prelimbic-hippocampal networks and poorer timing of neuronal firing in PL by hippocampal oscillations. These data might provide mechanistic insights into the functional dysmaturation of neuronal networks that accounts for cognitive deficits in psychiatric disorders of children experiencing early adversities.

Ensuring sufficient level of early stimulation, maternal care is crucial for brain development^34^,^35^. Consequently, its absence, even if transient, has been proposed to disrupt maturational processes and in the end, the behavioral performance. While the outcome of this form of ELS strongly depends on the time and frequency of separation^2^, profound cognitive deficits have been identified in adult humans and rodents experiencing maternal deprivation^3^,^4^,^36^. Our data reveal that the cognitive dysfunction is not present only in adults but emerges already at juvenile age and is both task- and sex-specific. On the one hand, male ELS rats were not able to recognize the new location and recency of an object, yet item recognition was intact. On the other hand, female rats were behaviorally less affected by maternal separation, since they were able to recognize the novelty and recency of an object, but not its location. The sex-specific impact of ELS on juvenile behavioral performance is in line with data from adult rodents and may relate to the larger overproduction and pruning in most brain regions^37^,^38^ as well as to the higher diurnal changes in cortisol levels of males when compared to females^39^. Remarkably, the mnemonic impairment seems to be age-dependent, since maternally-separated rats were able to recognize new objects at juvenile age, but failed to perform NOR at adulthood^4^,^40^. Similar age-related effects of ELS have been reported also for working-memory^25^.

**Figure 8:**
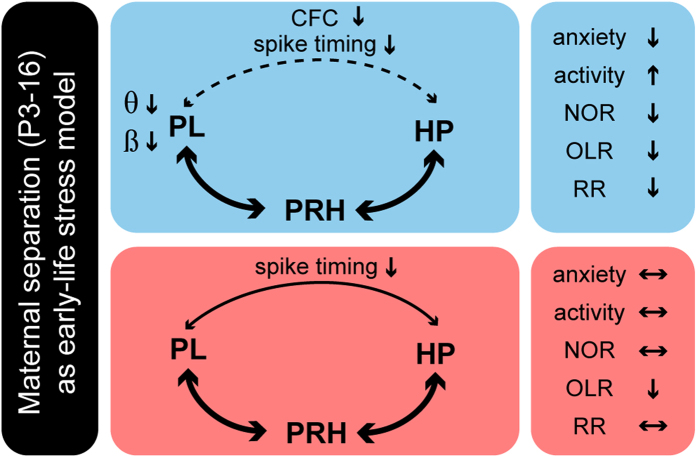
Network mechanisms underlying functional and behavioral deficits at juvenile age after ELS. Schematic diagram displaying changes in activity and communication within prelimbic-hippocampal-perirhinal networks as well as behavioral impairment of male (blue) and female (red) rats experiencing maternal separation during early life. Bold arrows represent intact communication between regions, whereas solid and dashed arrows indicate disrupted communication. The disruption is more severe in males (dashed line) than in females (solid arrow).

In line with the different types of information used to form judgements (e.g. relative familiarity of an object or location, where and when an object was encountered), the recognition memory is not a unitary process. Consequently, it does not result from activation of a single brain area, but from interactions within neuronal networks. A widely accepted model identified prefrontal-hippocampal coupling as a crucial factor for object location and recency recognition, but not for the novel object preference, which requires activation of perirhinal cortex^15^. The poorer performance in OLR and RR tasks of juvenile male rats with a history of ELS suggests that the juvenile prefrontal-hippocampal coupling is disrupted after maternal separation, as has been shown in adult rats after chronic stress^41^. Indeed, the level of prelimbic entrainment in theta and beta frequency range was decreased in ELS rats when compared with controls. Moreover, the weaker theta-gamma coupling within prefrontal-hippocampal networks and the decreased timing of prelimbic neurons by hippocampal theta in male ELS animals confirm the selective action of maternal deprivation on prefrontal-hippocampal networks of juvenile male rats (Fig. 8). In contrast to the hyper-excitability induced by ELS in developing hippocampal-amygdala circuits^42^, the level of activation within juvenile prefrontal-hippocampal networks seems to decrease as reflected by lower oscillatory power. These differences may reflect the distinct impact of maternal separation on brain circuitry. However, we cannot exclude that it relates to different behavioral states (awake vs. anesthetized) of investigated juvenile rats. It is very likely that under awake conditions, already the coupling within prefrontal-hippocampal-perirhinal networks of juvenile controls profoundly differs from that assessed under anesthesia-mimicked sleep conditions. Yet different attentional/alert states lead to increased heterogeneity of activity patterns and communication in non-anaesthetized states^43^.

How might ELS dampen the communication between PFC and HP? Besides activation of HPA axis and involvement of corticosteroids and CRH^4^,^44^, maternal separation causes major cellular and synaptic changes. Even if a causal link between cellular deficits and network dysfunction cannot be easily established^45^, these changes give firsts insights into the mechanisms of adversity. They have been extensively monitored in HP^10^,^46^,^47^ and, more recently, also in PFC^8^,^24^,^48^. Decreased cell density was detected in the adult HP after ELS^49^, yet different hippocampal subdivisions expressed different levels of vulnerability. Moreover, maternal separation led to dendritic atrophy and reduction of glutamatergic receptors^46^,^50^, some of these deficits being sex-specific^51^. The structural changes were strong enough to affect the functional plasticity (e.g. abnormal LTP) at adulthood^10^, especially in males^52^. However, as shown here, they seem to be insufficient for impairing the juvenile hippocampal activity. The incomplete magnitude of structural damage within re-organizing circuits at juvenile age or the compensatory action of extra-hippocampal generator of theta activity in CA1 may account for normal oscillatory patterns in HP. However, even if the hippocampal network activity was not affected, the synaptic transmission and communication of HP with other areas, especially with the PL, was significantly disturbed. During the entire development, PL tightly interacts with the CA1 area, the hippocampal drive timing the prelimbic firing and entrainment of local circuits in gamma rhythms^17^. Lower CFC and spike timing within prelimbic-hippocampal networks reflect weaker communication between juvenile PL and HP. Spine loss and atrophy of glutamatergic connections that link the two areas may act as structural substrate of functional deficits. Deprived of its critical drive during entire postnatal development, the overall maturation of PL might be impaired, as shown by the global decrease of theta and beta power of prelimbic oscillations.

Abundant literature highlights neuronal deficits in the adolescent and adult PL after ELS^8^,^24^,^48^,^53^. At structural level, maternal separation during the stress hyporesponsive period of development (P3–16), which is defined as a time-window of low circulating corticosterone level^54^, led to spine and dendritic atrophy in the PFC, especially in layer II/III^8^,^55^. Their impairment may contribute to the power decrease in PL, since these neurons are critical elements for the emergence of fast activity patterns within local circuits^56^. Besides stress-induced atrophy of connections and spines, two other mechanisms might underlie the excitability decrease and diminishment of network activation in PFC after ELS. First, maternal separation has been reported to cause loss of parvalbumin-positive interneurons in both males and females^25^,^48^, yet this deficit emerged later (e.g. at juvenile age) in males than females^57^. Interneurons control the local entrainment in beta-gamma of local circuits, their loss causing an imbalance of excitation-inhibition and consequently, abnormal network oscillations and coupling. Second, ELS mimicked by maternal separation causes long-lasting disruption of muscarinic receptor-mediated excitation of PFC^24^. We previously showed that during development muscarinic modulation strongly controls the level of prelimbic-hippocampal coupling^58^.

The impairment of processing within juvenile PFC is reflected not only by poorer performance in cognitive tasks, such as OLR and RR, but also by abnormal anxiety behavior in male rats with a history of ELS. The prelimbic region of PFC is particularly involved in fear- and anxiety-related processes. Our data revealed a decreased anxiety-like behavior in combination with hyper-activity in male rats that experienced maternal separation during the first two postnatal weeks. These findings are in contrast with previously reported increased anxiety of slightly older rats^8^. Whether this discrepancy results from different age or testing of behavior remains to be elucidated.

The present data uncover the network processes underlying the ELS-induced cognitive deficits during late development. They indicate that maternal separation differentially affects the behavioral performance of males and females. The behavioral deficits come together with substantial impairment of the functional coupling between brain areas. The identified processing deficits after ELS might represent the substrate of cognitive dysfunction in neuropsychiatric disorders related to emotional and physical neglect during early life^2^. In humans long duration adversity during childhood has been found to positively correlate with memory impairment^59^, yet only few studies focused on cognitive dysfunction of adolescents after maltreatment and neglect during childhood. Therefore, substantial effort needs to be put into the elucidation of long-term consequences of adversity during early life.

## Materials and Methods

All experiments were performed in compliance with the German laws and the guidelines of the European Community for the use of animals in research and were approved by the local ethical committee of the city Hamburg (94/08, 111/12, 132/12). All efforts were made to minimize animal suffering and the number of animals used. Pregnant Wistar rats were obtained at 14–17 days of gestation from the animal facility of the University Medical Center Hamburg-Eppendorf, housed individually in breeding cages with a 12 h light/12 h dark cycle and fed *ad libitum*.

### Maternal separation procedure

On postnatal day (P) 1 litters were culled to 6–10 pups in a constant sex ratio (1:1) to avoid sex-based maternal behavioral biases^60^,^61^,^62^. Both male and female pups were included in the study. Litters were randomly assigned to either CON or ELS group. Pups of ELS group experienced daily separation from the mother and siblings from P3 to 16 for 3 h (11 am–2 pm). They were transferred to a humidity-controlled adjacent room and kept individually in cages supplied with bedding material from the home cage (Fig. 1). To maintain their physiological body temperature the cages were placed on a heating pad at 34 °C. Pups classified as CON remained with their mother and siblings, but experienced the same amount of handling as ELS pups.

### Recording protocols

Extracellular recordings were simultaneously performed from the PL (n = 38 rats, 2–2.5 mm anterior to bregma and 0.2–0.7 mm from the midline), CA1 area of intermediate HP (n = 38 rats, 6 mm posterior to bregma and 6 mm from the midline) and PRH (n = 27 rats, 6 mm posterior to bregma and 8 mm from the midline) of the majority of behaviorally-tested P21–22 male and female rats using experimental protocols previously described^17^. Under light urethane-anesthesia (1 g/kg; Sigma-Aldrich, Taufkirchen, Germany), the head of the pup was fixed into the stereotaxic apparatus (Stoelting, Wood Dale, IL) using two metal bars fixed with dental cement on the nasal and occipital bones, respectively (Fig. 1). The bone over the PFC, HP and PRH was carefully removed by drilling holes of less than 0.5 mm in diameter. Removal of the underlying dura mater by drilling was avoided, since leakage of cerebrospinal fluid or blood damps the cortical activity and single neuronal firing (I. Hanganu-Opatz, personal observations). The body of the animals was kept at a constant temperature of 37 °C by placing it on a heating blanket. After a 20–40 min recovery period, multielectrode arrays (Silicon Michigan probes, for PL: A1 × 16-3 mm-100–703, for HP: A1 × 16-5 mm-50–703, for PRH: A1 × 16-5 mm-150–703, NeuroNexus Technologies, Ann Arbor, MI) were inserted perpendicularly to the skull surface into the PL until a depth of 3 mm, at 15° from the vertical plane into the HP at a depth of 2–2.5 mm and at 15° from the vertical plane into the PRH at a depth of 2.5–3 mm. The electrodes were labeled with DiI (1,1′-dioctadecyl-3,3,3′,3′-tetramethyl indocarbocyanine, Invitrogen, Darmstadt, Germany) to enable the reconstruction of their tracks in the PL, HP and PRH in post-mortem histological sections. One silver wire inserted into the cerebellum served as ground electrode.

Recordings (0.1 Hz–9 kHz) of the local field potential (LFP) and multiple unit activity (MUA) were performed using one-shank 16-channel Michigan electrodes (impedance 0.4–0.7 MΩ). The recording sites were separated by 50 (for HP), 100 (for PL) or 150 μm (for PRH) in vertical direction. Both LFP and MUA were recorded for at least 3600 s at a sampling rate of 32 kHz using a multi-channel extracellular amplifier (Digital Lynx 4 S, Neuralynx, Bozeman, MO) and the corresponding acquisition software (Cheetah). After histological confirmation of the correct electrode position one recording site/region was selected for further LFP analysis. For PFC one recording site with high firing and prominent LFP oscillations confined to the prelimbic subdivision was considered. For HP one channel below the LFP reversal over str. pyramidale of CA1 was selected for analysis. For SUA analysis all recording sites confined to the brain region of interest were considered.

### Behavioral analysis

The behavior of ELS and CON rats was investigated in parallel. To minimize the influence of circadian rhythms, all behavioral tests were conducted during the same phase of circadian cycle (i.e. light phase). One investigator performed all behavioral testing to prevent inter-observer variability due to different handling of pups.

The exploratory behavior and recognition memory of CON and ELS rats were tested at juvenile age (P17–20) using experimental protocols previously developed in our lab^19^. Briefly, all behavioral tests were conducted in a circular black arena, the size of which (D: 46 cm, H: 30 cm) maximized exploratory behavior^63^, while minimizing incidental contact with testing objects. The objects used for testing of novelty recognition were six differently shaped and textured, easy to clean items that were provided with magnets to fix them to the bottom of the arena. Object sizes (H: 5–9 cm, diameter: 3–4.5 cm) were smaller than twice the size of the rat and did not resemble living stimuli (no eye spots, predator shape). The objects were positioned at 15.5 cm from the borders and 7.5 cm from the center of the arena (Fig. 1). After every trial the objects and arena were cleaned with 70% ethanol to remove all odors. A black and white CCD camera (VIDEOR TECHNICAL E. Hartig GmbH, Roedermark, Germany) was mounted 90 cm above the arena and connected to a PC via PCI interface serving as frame grabber for video tracking software (Video Mot2 software, TSE Systems GmbH, Bad Homburg, Germany).

#### Exploratory behavior in the open field

Pre-juvenile rats (P17) were allowed to freely explore the testing arena for 10 min. During this time grooming, rearing and wall-rearing were quantified in their occurrence and duration. Additionally the floor area of the arena was digitally subdivided in 8 zones (4 center zones and 4 border zones) using the zone monitor mode of the VideoMot 2 analysis software (VideoMot 2, TSE Systems GmbH). The time spent by pups in center and border zones as well as the running distance and velocity were analyzed.

#### Novelty recognition paradigms

All protocols for assessing item, place and temporal order recognition memory in P18–20 rats consisted of familiarization and testing trials^9^. During the familiarization trial each rat was placed into the arena containing two identical objects and released against the center of the opposite wall with the back to the objects. After 7 min of free exploration of objects the rat was returned to a temporary holding cage. Subsequently, test trials for novel object recognition (NOR), object location recognition (OLR) and recency recognition (RR) were performed after a delay of 5 min post-familiarization. During NOR P18 pups were allowed to investigate one familiar and one novel object with different shape, color and texture (Fig. 2Ai). In the OLR task, which was achieved at P19, the position of one of the objects was changed (Fig. 2Bi). In the RR task two familiarization trials with two different pairs of objects were achieved and were separated by a delay of 30 min. During the testing trial one object used in the first and one used in the second more recent familiarization trial were placed in the arena at the same positions as during the familiarization trials (Fig. 2Ci).

All trials were video-tracked and the analysis was performed using the Video Mot2 analysis software. The object recognition module of the software was used and a 3-point tracking method identified the head, the rear end and the center of gravity of the rat (Fig. 1). Digitally, a circular zone of 2 cm was created around each object and every entry of the head point into this area was considered as object interaction. Climbing or sitting on the object, mirrored by the presence of both head and center of gravity points within the circular zone, were not counted as interactions. Rats demonstrating insufficient task performance (e.g. non-interaction with objects in the familiarization trials) were excluded from analysis (n = 5). Discrimination ratio was calculated by dividing the time that pups additionally spent investigating the novel object by the total time of interaction with the two (novel and familiar) objects. Values were calculated using the first 2 min (NOR and OLR) and 1 min (RR), as controls showed highest preference for the new object during these time intervals.

### Histology

Juvenile rats were deeply anesthetized with 10% ketamine (aniMedica, Senden-Bösensell, Germany)/2% xylazine (WDT, Garbsen, Germany) in NaCl (10 μl/g body weight, i.p.) and perfused transcardially with 4% paraformaldehyde dissolved in 0.1 M phosphate buffer, pH 7.4. The brains were removed and postfixed in the same solution for 24 h. Subsequently, fixed brains were sectioned in the coronal plane at 100 μm using a vibrating blade microtome (Vibratome, Bannockburn, USA). For the reconstruction of DiI-labeled electrode tracks in PL, HP and PRH, the slices were stained with Hoechst 33342 dye (1:1000, Invitrogen, Eugene, OR), air dried, coverslipped with Fluoromount and examined using the 380 and 568 nm excitation filters of the Imager M1 microscope (Zeiss, Oberkochen, Germany).

### Data Analysis

Data were imported and analyzed off-line without knowledge on sex and group identity of rats (CON or ELS) using custom-written tools in Matlab software, version 8.1 (Mathworks, Natick, MA).

#### LFP analysis

In a first step, the raw wide-band signal was processed by filtering (<300 Hz) with a 3^rd^-order Butterworth zero-phase filter and downsampling to 3255 Hz. Power spectral density estimates were calculated using the MATLAB function ‘pwelch’, with a 10 s window and 50% overlap. These power spectral estimates were used to calculate band power for different frequency bands. Maximal coherence as spectral measure of correlation between two signals across frequencies was calculated using the magnitude-squared coherence function ‘mscohere’ (MATLAB) based on Welch’s averaged modified periodogram method (with 50% overlap and 5 s time window). Theta-gamma cross-frequency coupling (CFC) was calculated as previously described^64^ by comparing the modulation indices (MI) averaged for each group (CON, ELS). Briefly, signals from two brain areas were filtered in both frequency bands (theta: 4–12 Hz, gamma: 30–100 Hz) and processed by computation of Hilbert transform to obtain their amplitude and phase. The normalized MI was determined as the deviation between an empirical amplitude distribution and a uniform distribution. MI = 1 correspond to max phase-amplitude coupling, whereas MI = 0 indicates a uniform distribution of phase-amplitude values, i.e. no phase-amplitude coupling. Only MI values significantly different from those calculated for shuffled amplitude signal (time-window of shuffling 5 s) were considered.

#### Spike sorting and analysis

To obtain single unit activity (SUA) the LFP was processed as following. The wideband-signal was filtered (>400 Hz) using a 3^rd^-order Butterworth zero-phase filter. Negative peaks exceeding 6 times the SD of the signal were detected and sorted into similar waveform shapes as previously described^65^ using the Offline Sorter software (Plexon, Dallas, TX). For depicting the valid waveforms in 2D/3D space a combination of features, including the first three principal components, was chosen. Shapes of detected waveforms were visually inspected to exclude background noise. A group of similar waveforms was considered as being generated from a single neuron if it defined a discrete cluster in a 2D/3D space and exhibited a refractory period (>1 ms) in the interspike interval (ISI) histograms. The quality of separation between identified clusters was assessed by four different statistical measurements: the classical parametric F statistic of multivariate analysis of variance (MANOVA), the J3 and PseudoF (PsF) statistics and the Davies-Bouldin validity index (DB)^66^. The values of statistical testing ranged between 1.74 and 25.04 for MANOVA, 0.39 and 16.2 for J3, 8.16 × 10^2^ and 2.88 × 10^5^ for PsF, and 0.17 and 0.71 for DB. The phase-locking between spikes and LFP was assessed using a previously described algorithm based on Rayleigh Z-statistic^31^. If the firing of a neuron is modulated by the theta rhythm then its phase over the theta cycle is not uniformly distributed. Phases of hippocampal and prefrontal theta rhythm were determined using the Hilbert transform (phase of 0 refers to the peak and phase of π/−π refers to the trough of the cycle). Because of its independence of spike number the strength of phase-locking was computed using pairwise phase consistency (PPC)^32^,^33^. PPC is defined by the average pairwise circular distance, that is calculated as follows


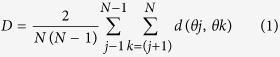


where *d* is the absolute angular distance of the two phases *θj* and *θk*, at which two spikes occur. N is the number of spikes. PPC is normalized as the following:


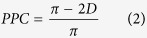


PPC shows negative values if spikes are uniformly distributed.

#### Statistics

Statistical analyses were performed with SPSS Statistics version 22 (SPSS GmbH) and MATLAB. Values were tested for normal distribution by the Kolmogorov-Smirnov test, except when their low number (n < 10) precluded reliable testing. For normally distributed values paired or unpaired t-test were used. For low number of values or not normally distributed values either Mann-Whitney U test or Wilcoxon test were used. Sex, group and region specific effects were tested using a two-/three-way ANOVA, using Scheffé post-hoc tests for region specific results. For some analyses Bonferroni correction was used to adjust the significance level. Statistical testing of phase locking was performed using t-test for the mean resultant vector length. Significance levels of p < 0.05 (*), p < 0.01 (**) or p < 0.001 (***) were detected. Data in the text are presented as mean ± standard error of the mean (SEM) and displayed as bar diagrams.

## Additional Information

**How to cite this article:** Reincke, S. A. J. and Hanganu-Opatz, I. L. Early-life stress impairs recognition memory and perturbs the functional maturation of prefrontal-hippocampal-perirhinal networks. *Sci. Rep.* **7**, 42042; doi: 10.1038/srep42042 (2017).

**Publisher's note:** Springer Nature remains neutral with regard to jurisdictional claims in published maps and institutional affiliations.

## Figures and Tables

**Table 1:** Power of oscillatory activity recorded in different frequency bands from the PL and HP of male and female controls and ELS rats. Values are given in dB as mean ± SEM.

		male		t-test		female		t-test	
		CON	ELS	t	p	CON	ELS	t	p
PL	4–12 Hz	25.38 ± 0.37	23.31 ± 0.61	2.96	0.009	23.93 ± 0.21	24.05 ± 0.37	−0.31	0.76
	12–30 Hz	14.36 ± 0.39	12.60 ± 0.48	2.88	0.01	13.58 ± 0.32	13.26 ± 0.26	0.74	0.47
	30–100 Hz	4.84 ± 0.42	4.04 ± 0.34	1.46	0.16	4.82 ± 0.38	4.66 ± 0.44	0.27	0.79
HP	4–12 Hz	24.53 ± 0.49	23.21 ± 0.52	1.83	0.08	23.06 ± 0.28	23.07 ± 0.33	−0.02	0.98
	12–30 Hz	15.93 ± 0.51	14.91 ± 0.37	1.59	0.13	14.65 ± 0.39	15.15 ± 0.31	−0.95	0.36
	30–100 Hz	7.84 ± 0.50	7.72 ± 0.21	0.22	0.83	7.46 ± 0.40	8.28 ± 0.22	1.60	0.13
PRH	4–12 Hz	23.75 ± 0.80	24.34 ± 0.43	−0.86	0.41	22.94 ± 0.40	22.80 ± 0.58	0.20	0.84
	12–30 Hz	14.20 ± 0.97	14.27 ± 0.41	−0.08	0.94	13.54 ± 0.52	13.11 ± 0.61	0.53	0.61
	30–100 Hz	4.15 ± 0.45	4.16 ± 0.37	−0.03	0.98	3.60 ± 0.38	3.43 ± 0.31	0.35	0.73
